# Renal Replacement Therapy in Acute Kidney Failure due to Rhabdomyolysis

**DOI:** 10.1155/2012/603849

**Published:** 2012-04-17

**Authors:** G. Maggi, F. Quinteros Hinojosa, M. J. Villagran, E. Guasch Arévalo, F. Gilsanz Rodríguez

**Affiliations:** Department of Anesthesiology and Resuscitation, La Paz University Hospital, Paseo de la Castellana 261, 28046 Madrid, Spain

## Abstract

Rhabdomyolysis is a syndrome caused by skeletal muscle cells destruction which can occur for many reasons, including prolonged immobilization. The main complication of the syndrome is the development of acute renal failure. Rhabdomyolysis and myoglobinuria are responsible for approximately 5% of all causes of acute renal failure in the USA. The cause of rhabdomyolysis is often multifactorial, and approximately 8–20% of such patients develop myoglobinuric acute renal failure.

## 1. Introduction

Rhabdomyolysis is characterized by muscle cell destruction and the subsequent leakage of muscle cell contents, including electrolytes, sarcoplasmic myoglobin, and other proteins into the circulation [1]. Acute kidney injury is a potential complication of severe rhabdomyolysis [2], and the prognosis is much worse if renal failure develops when the main cause is any trauma [3, 4]. In spite of the fact that conventional hemodialysis filters do not remove myoglobin, hemodiafiltration with super-high-flux dialyzers may be effective [5]. Our aim is to report a case of acute kidney injury due to rhabdomyolysis in which we used continuous venovenous hemodiafiltration (CVVH) in the intensive care unit of a tertiary university hospital.

## 2. Case Presentation

An 82-year-old woman, independent as regards activities of daily life, was admitted into the emergency department due to accidental fall at home with consecutive immobilization for a period of approximately 12 hours. Her medical history includes hypertension, dyslipidemia, non-insulin-dependent diabetes, and mild chronic renal insufficiency (baseline creatinine 1.4 mg/dL).

The accidental fall caused right femur periprosthetic supracondylar fracture, which is pending surgery. Blood sample analysis at admission was the following: creatinine 8.74 mg/dL, urea 210 mg/dL with anuria compatible with kidney injury according to RIFLE criteria [6], and the patient was transferred to the intensive care unit (ICU).

At the time of admission to the ICU, blood sample analysis showed the following: creatinine 8.76 mg/dL, urea 202 mg/dL, and potassium 6.4 mEq/L, and arterial blood gas analysis showed metabolic acidosis: pH 7.02, −24.6 base excess, serum bicarbonate 4.3 mEq/L, partial pressure of oxigen 79 mmHg and partial pressure of carbon dioxide 16.9 mmHg of creatine phosphokinase (CK) 9475 U/L, with oliguria. Continuous venovenous hemofiltration (CVVHF) with (Edwards Aquarius, Irvine-based Edwards Lifesciences Corp. Lone Peak Parkway, Draper, UT 84020, USA Irvine, CA, USA) 1,2 polysulphone m2 membrane was started with the sequent parameters: 180 mL/min blood flow, predilution flow of 1000 mL, postdilution of 1000 mL, removal of 100 mL/hour, prefilter pressure of 180 mmHg and effluent pressure of 70 mmHg, with prefilter/effluent relationship 30/70. Due to the fact that the patient was breathing with difficulty and was progressively showing clinical and analytical deterioration, a chest radiograph was performed, which showed bilateral infiltrates compatible with distress syndrome with right-lower lobe consolidation added. Endotracheal intubation and mechanical ventilation was conducted the second day at ICU. Blood culture, bronchoalveolar lavage, culture of the fracture site, and sputum cultures were conducted. The last one showed *Staphylococcus aureus* and *Neisseria meningitides* colonization and the right femur periprosthetic sample showed *Pseudomonas aeruginosa*, so meropenem 1 g every 12 hours was administered. The patient was hemodynamically unstable, and thus she was invasively monitored, requiring inotropic support with norepinephrine presenting septic shock with acute circulatory failure with persistent arterial hypotension despite adequate fluid resuscitation. Radiological and clinical improvement allowed extubation, and due to creatinine level improvement (1.03 mg/dL), urea (59 mg/dL) and CK (137 U/L) it was decided to withdraw CVVHF. The patient had a favorable outcome with spontaneous dieresis and was eupneic without respiratory support, hemodynamically stable, and afebrile.

Within a week of their stay in our critical care unit, scheduled surgery was performed for her right femur periprosthetic supracondylar fracture, with spinal anesthesia without incidents. After surgery she moved back to our critical care unit, where she remained eupneic and hemodynamically stable with spontaneous diuresis and good renal function (creatinine 0.92 mg/dL), so after 12 hours in RU, she was discharged to ward.

## 3. Discussion

Acute renal failure associated with myoglobinuria is the most serious complication of traumatic and nontraumatic rhabdomyolysis, which can be life threatening [7]. Acute kidney injury as a complication of rhabdomyolysis is quite common, representing about 7 to 10% of cases of acute kidney injury in the United States [4].

Myoglobin is a 17.8 kDa protein of molecular mass that is freely filtered by the glomerulus, entering tubular epithelial cells by endocytosis, where it is metabolized, appearing in the urine only when the renal threshold of 0.5 to 1.5 mg/dL of myoglobin is exceeded and is grossly visible as reddish-brown (“tea-colored”) urine when serum myoglobin levels reach 100 mg per deciliter [4]. Although the exact mechanisms by which rhabdomyolysis affects the rate of glomerular filtration are not clear, experimental evidence suggests the following mechanisms: (1) intrarenal vasoconstriction, (2) direct and ischemic tubular injury, and (3) tubular obstruction [8]. Myoglobin is concentrated along the renal tubules, a process accentuated by volume depletion and renal vasoconstriction. This interaction with the Tamm-Horsfall produces protein precipitation, a process favored by acidic urine; tubule obstruction occurs mainly in the distal tubules, and direct cytotoxicity occurs mainly in the proximal tubules [9]. Myoglobin seems to have no marked nephrotoxic effect on the tubules unless the urine is acidic. Myoglobin is a heme protein; it contains iron, as ferrous oxide, which is necessary for the binding of molecular oxygen. However, molecular oxygen can promote the oxidation of ferrous to ferric oxide, thus generating a hydroxyl radical. This oxidative potential is countered by effective intracellular antioxidant molecules. However, cellular release of myoglobin leads to uncontrolled leakage of reactive oxygen species, and free radicals cause cellular injury. It has been suggested that heme and free iron-driven hydroxyl radicals are critical mediators of tubule damage owing to the protective effects of deferoxamine (an iron chelator) and glutathione [10].

There is no defined threshold value of serum creatine kinase above which the risk of acute kidney injury is markedly increased [11]. There was a very weak correlation between peak serum creatine kinase and the incidence of acute renal failure [12].

Myoglobin is the real pathogenic factor in rhabdomyolysis and acute renal failure, but rarely measured directly in urine or plasma [13]. The measurement of serum myoglobin has low sensitivity for the diagnosis of rhabdomyolysis [14].

Patients with rhabdomyolysis associated with acute renal failure commonly present clinical symptoms of volume depletion due to the kidnapping of water in the injured muscles. Therefore, it is essential to implement aggressive fluid replacement; it may require up to 10 liters of fluid per day, depending on the severity of the rhabdomyolysis [15]. Clinical benefits of alkalinization as compared with simple volume repletion are not firmly established [16, 17].

Some authors propose to consider renal replacement therapy in rhabdomyolysis if there is resistant hyperkalemia of more than 6.5 mmol per liter that is symptomatic (assessed by electrocardiography), rapidly rising serum potassium, oliguria (<0.5 mL of urine per kilogram per hour for 12 hours), anuria, volume overload, or resistant metabolic acidosis—pH < 7.1) [18]. Common techniques of dialysis have shown a limited capacity for removal of circulating myoglobin [19]. In a recent article, Naka and colleagues [5] have proposed the use of a membrane of super-high-flow continuous hemofiltration with promising results.

Why are extracorporeal techniques ineffective in removing myoglobin? There are several reasons that depend on the nature of the molecule, on its distribution in the organism, on the mechanism of solute transport, and on the structure of the membrane in the extracorporeal technique.

Myoglobin is 17 kDa PM, caries an electrical charge, and can be considered as a solute with a radius larger than expected. In these circumstances, it has a very low diffusion coefficient, thus requiring transport by convection, but also has a spherical size so it is likely to be rejected by the membrane pores. The standard cellulosic membranes are virtually impermeable to the molecule; therefore, high-flux membranes should be used [20].

The limitation of the therapy as a high-flux hemofiltration is the presence of low sieving coefficient for myoglobin filtration; even a high-volume hemofiltration or pulse high-volume hemofiltration may be inefficient [21].

The solution proposed by Naka and colleagues [5] seems to be feasible and effective in removing myoglobin. The use of a continuous technique with a high-flux membrane hyperpermeable to myoglobin seems to guarantee the elimination of it [16]. One possible limitation is represented by albumin leakage, which should be rigorously tested and evaluated [22].

In our case, acute renal failure with CVVH management, no myoglobin levels were measured but its use showed progressive improvement in renal function with decreased CK levels after 5 days of use, decreasing creatinine levels from 8.76 to 1.03 mg/dL and CK levels of 9475 to 137 U/L.

In conclusion, the use of hyperpermeable membranes in CVVH could represent a new approach to treating acute rhabdomyolysis, because it provides not only an efficient renal replacement, but also a protective effect with fast and efficient removal of circulating myoglobin.

However, the available evidence comes from few case series, and the effect on the results is unknown. In addition, some studies have shown that the half-life of serum myoglobin did not differ significantly between patients who are treated conservatively and those who receive CVVH. So, due to the fact that randomized controlled trials have not been carried out yet, the preventive use of CVVH in acute renal failure secondary to rhabdomyolysis cannot be recommended.
